# Nest-Gallery Development and Caste Composition of Isolated Foraging Groups of the Drywood Termite, *Incisitermes minor* (Isoptera: Kalotermitidae)

**DOI:** 10.3390/insects7030038

**Published:** 2016-07-22

**Authors:** S. Khoirul Himmi, Tsuyoshi Yoshimura, Yoshiyuki Yanase, Masao Oya, Toshiyuki Torigoe, Masanori Akada, Setsuo Imadzu

**Affiliations:** 1 Research Institute for Sustainable Humanosphere (RISH), Kyoto University, Gokasho, Uji, Kyoto 611-0011, Japan; tsuyoshi@rish.kyoto-u.ac.jp; 2 Research Center for Biomaterials, Indonesian Institute of Sciences (LIPI), Jl. Raya Bogor km. 46 Cibinong, Bogor 16911, Indonesia; 3 Graduate School of Agriculture, Kyoto University, Sakyo-ku, Kyoto 606-8502, Japan; yanase@h3news1.kais.kyoto-u.ac.jp; 4 Oya Shiroari Giken, 438-1 Oaza-ichiya, Nachikatsuura-cho, Higashimuro-gun, Wakayama 649-5141, Japan; oyasiroarigiken@za.ztv.ne.jp; 5 Nara National Museum, 50 Noborioji-cho, Nara 630-8213, Japan; torigoe@narahaku.go.jp; 6 Kyushu National Museum, 4-7-2 Ishizaka, Dazaifu, Fukuoka 818-0118, Japan; akada004001@gmail.com (M.A.); imazu@kyuhaku.jp (S.I.)

**Keywords:** nest-gallery development, X-ray computed tomography, caste composition, *Incisitermes minor*

## Abstract

An X-ray computed-tomographic examination of nest-gallery development from timbers naturally infested by foraging groups of *Incisitermes minor* colonies was conducted. This study documents the colonization process of *I. minor* to new timbers and how the isolated groups maintain their nest-gallery system. The results suggested that development of a nest-gallery within a suitable wood item is not random, but shows selection for softer substrate and other adaptations to the different timber environments. Stigmergic coordinations were expressed in dynamic changes of the nest-gallery system; indicated by fortification behavior in sealing and re-opening a tunnel approaching the outer edge of the timber, and accumulating fecal pellets in particular chambers located beneath the timber surface. The study also examines the caste composition of isolated groups to discover how *I. minor* sustains colonies with and without primary reproductives.

## 1. Introduction

The introduction of X-ray computed tomography (CT) as a non-destructive approach to analyzing termite nests has brought us a better understanding of the nesting biology of this cryptic insect. Fuchs et al. [1] were the first to introduce CT as a useful tool for providing a three-dimensional (3D) view of the hidden gallery system of drywood termite *Cryptotermes secundus*, though at a low-resolution display. Perna et al. presented detailed images of gallery networks [2] of *Cubitermes* sp. and used them to analyze their topological defense strategy [3] and efficiency [4], while Himmi et al. [5] used X-ray CT to analyze the initial structure of the royal chamber of the western drywood termite, *Incisitermes minor*.

X-ray CT is a proven technique to analyze the nest architecture of one-piece nesters, i.e., termite colonies that forage and nest in a single piece of wood [6]. Other physical methods, such as casting [7], by injecting a thin slurry of orthodontal plaster into the nest; or wood dissection [8,9,10] cannot be used to map the whole system of a nest-gallery. Himmi et al. [11] analyzed the timbers with an established nest-gallery system of *I. minor*, and observed that the interactions between the colony and environment, particularly the anatomical properties of the wood fibers and growth rings, generate a distinctive and unique foraging pattern which leads to selective excavation. However, the study did n capture the in situ development of a nest-gallery system, which maps the development from the initial stage of the nest-founding activity.

Two kinds of nest-founding activities have been identified in drywood termites [5]: (1) from nuptial flight by pairing dealate reproductives; or (2) from nest-gallery extension through colony foraging to a new piece of adjacent timber [8]. In this paper, we present a case study of nest-gallery development from timbers naturally infested by an *I. minor* colony through foraging activity. The study was intended to capture the colonization process of foraging groups of *I. minor* in previously unoccupied timber and how the groups maintain their nest-gallery system. The study also examines the caste composition of isolated groups in search of a better understanding of how one-piece nester types sustain their colonies.

## 2. Materials and Method

### 2.1. Wood Specimen and Experimental Setup

We set up a field experiment on the nest-founding activity of *I. minor* in Wakayama prefecture, Japan, in August 2012 using two commercial timbers, Sitka spruce (*Picea sitchensis* Bong. Carriere) and sugi (*Cryptomeria japonica* D. Don). The timbers had dimensions of 50 (*R*) × 50 (*T*) × 1000 mm (*L*) and were made up of a combination of sapwood and heartwood portions. The timbers were laid in the attic areas of four highly-infested houses. A total of 10 sugi (S) timbers and 15 spruce (P) timbers were placed in each house, arranged at five different test areas. Each test area consisted of five pieces of timbers, with a “P-S-P-S-P” arrangement and narrow gap (~1 cm) between the timbers. The timbers were arranged at random orientations, without considering whether tangential sections, radial sections, sapwood parts, and heartwood parts faced any particular direction.

### 2.2. Monitoring

Annual monitoring was conducted in November, around two months after the swarming season of *I. minor* in Wakayama. All of the infested timbers were brought back to the laboratory and were kept at controlled temperature and humidity (28 ± 20 °C; RHs 80% ± 10%). Two nest-founding strategies were observed (Table 1): first, through nuptial flight by a pair of dealate reproductives; and second, through colony activity in extending the nest-gallery to the adjacent bottom surface of the timber. Three timbers infested by colony activity (Table 1) are presented in the present study. Those timbers were collected from the same test area, but at different times. Details of the infestation can be found in Table 2.

### 2.3. Analysis

#### 2.3.1. X-Ray CT Scanning and Data Analysis

The timbers were analyzed by an X-ray CT Scanning Machine (Y.CT Modular320 FPD, YXLON International GmbH, Hamburg, Germany) at Kyushu National Museum to visualize the nest-gallery system. Spruce A was subjected to biannual analysis (from 2012 to 2014) to observe the in situ gallery development, while the other two timbers were scanned only once, in November 2014. When it was collected, Spruce A had only one chamber and was at the very beginning of new nest-gallery extension [5]; therefore, we were able to record the development of the nest-gallery by the *I. minor* colony by continuously scanning the timber. Spruce B and Sugi already had an extensive nest-gallery system when it was found; therefore, it was very difficult to follow the development of a nest-gallery system in those timbers.

The scanning analysis was conducted in vertical measurement by 320-kV X-ray source (2.0 mA) with a 400 mm × 400 mm digital flat-panel refractive index detector (RID) (YXLON International GmbH, Hamburg, Germany) in the dynamic range of 16-bit, pixel pitch of 20 µm, and 1024 × 1024 pixel resolution. The timbers were put on a rotary table that continuously rotates during the scanning analysis while, at the same time, the X-ray tube and the detector were simultaneously moved vertically and downward along the entire length of the timbers. The X-ray CT data were stored in files containing two-dimensional (2D) image stacks (*.raw image file), with a digital thickness of 0.3 mm. Each file of 2D image stacks corresponds to a single 2D image-slice of the timbers. CT data analysis was performed by rendering 2D CT image data into 3D images using volume graphics software (VGStudio MAX 2.1, Volume Graphics GmbH, Hamburg, Germany).

#### 2.3.2. Colony Examination

The timbers were kept in the laboratory to allow termite colonies to develop and extend the nest gallery system. The timbers were opened in March 2016 (Table 2), and all termites were carefully extracted from each timber. The timbers were cut into pieces no longer than 100 mm, and termites that fell from the timber were collected. The pieces of timber were carefully chopped and all the termites were carefully collected and held. The composition of colony caste was examined with a KEYENCE VHX-5000 digital microscope (Keyence Corp., Osaka, Japan).

## 3. Results

**Figure 1:**
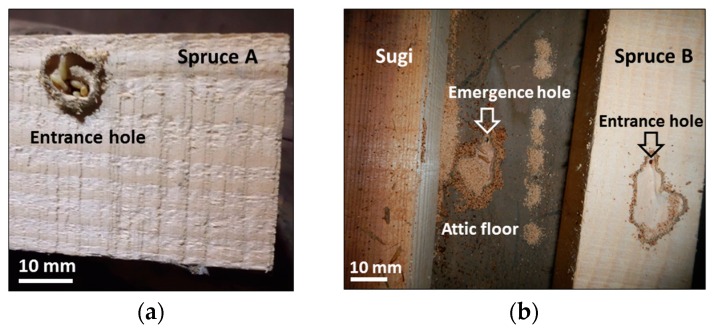
Nest-gallery extension from natal colony to adjacent timber activity by an *I. minor* colony. (**a**) Entrance hole in Spruce A timber; and (**b**) *I. minor* colony emerged from the attic floor to attack the adjacent bottom surface of Spruce B and Sugi timbers.

The three timbers—Spruce A, Spruce B, and Sugi—were infested by an *I. minor* colony in a similar process: the infestation was mediated by foraging activities of individuals that had emerged from their natal nest. In both spruce timbers, the colony emerged from the attic floor to attack the adjacent bottom-surface of the timbers (Figure 1). An ample entrance hole was excavated by the colony, surrounded by cement pellets to connect it with the emergence hole of the natal colony. In the Spruce A timber, the entrance hole was excavated on a tangential section of the sapwood, while in the Spruce B timber, it was excavated on a tangential section of the heartwood (Table 2). A group of termites was engaged in excavation activities when the timbers were collected (Figure 1). The entrance hole in the Sugi timber could not be located. However, many holes sealed with fecal pellets were identified all over the sapwood surface.

### 3.1. Nest-Gallery Development in Spruce A Timber

**Figure 2:**
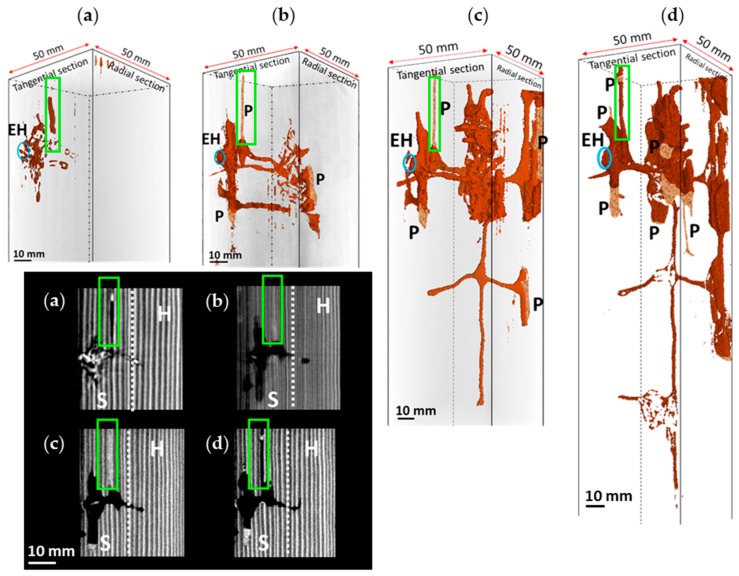
The 3D and 2D CT images of nest-gallery development of Spruce A timber: (**a**) initial scan; (**b**) half-year later; (**c**) one year later; and (**d**) one and a half years later. Two-dimensional radial-section images show dynamic change of the nest-gallery system, indicated by the sealing and re-opening of a tunnel gallery (green rectangles). In 3D images, the presence of termites is indicated by the uncolored area inside the nest-gallery. In the 2D CT images, the gray value of a pixel corresponds to an index of density: the lighter the color, the denser the area [11]. Springwood and summerwood are indicated by the darker and lighter colors in the images, respectively. EH, entrance hole; S, sapwood; H, heartwood; P, fecal pellets.

**Figure 3:**
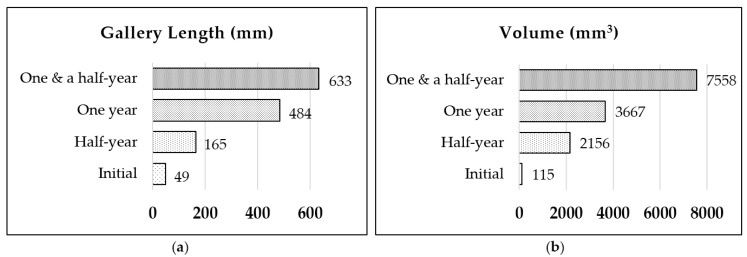
The properties of nest-gallery development in Spruce A timber: (**a**) gallery length (mm); and (**b**) volume (mm^3^).

Figure 2 presents 3D and 2D CT images of biannual development of the nest-gallery system in Spruce A timber, while the length and volume properties are shown in Figure 3. The initial scan (Figure 3a) showed that the timber had a newly-excavated chamber. This chamber was an extension for infesting a new timber, by the fact that the entrance hole was adjacent to the emergence hole of the natal nest from attic floor. A detailed description of the initial structure of the chamber was reported by Himmi et al. [5]. Five exploratory tunnels were extended from the chamber in five different directions (Figure 2a, 3D image), which may constitute an environmental assessment performed by the isolated group before it further extended the nest-gallery.

A half-year later, the group had extended the nest gallery by excavating another chamber through one of the exploratory tunnels that had been excavated perpendicularly to the axial system (Figure 2a,b, 3D images). Both chambers were still in the sapwood part of the Spruce A timber. One year later, the nest-gallery system comprised numerous chambers and interconnected tunnel-galleries (Figure 2c, 3D image) and had expanded throughout both the sapwood and heartwood parts of the timber. Chambers were constructed as spacious cells around an annual growth ring, or involving several growth rings when the width of the growth ring was narrow (<2 mm). The tunnels had circular cross-sections, and were constructed very narrowly and primarily along the springwood. The diameters of the tunnels were restricted in size (~2 mm), a gap that would only allow a single termite passage through. One and a half years later, the nest-gallery had more extensive chambers and tunnel galleries (Figure 2d, 3D image). The presence of termites is indicated by an uncolored area inside the nest-gallery, and the pellets are located in certain chambers just beneath the timber surface. The CT images indicated that termites aggregated in particular chambers (Figure 2a–d). The group seemed to keep moving together to new chambers (Figure 2b–d) and to leave the previous chambers empty. Some individuals were observed at the edge of the tunnel galleries (Figure 2d), which may be related with tunneling activity.

In 2D radial section images, we observed the dynamic change of the nest-gallery system. One of the exploratory tunnels from the first chamber was extended parallel to the longitudinal axis, and reached the outer edge of the timber (Figure 2a, green rectangle). A half-year later, the tunnel was sealed by cement pellets and the tunnel remained sealed at the one-year scan (Figure 2b,c, green rectangle). Interestingly, the sealed tunnel was re-accessable, as we found that termites had re-opened the tunnel after one and a half years (Figure 2d, green rectangle), leaving tiny cement pellets to close the tunnels to the outside environment.

The development of the length and volume (Figure 3) had a pattern that indicated the basic strategy of the isolated group to extend the nest-gallery system in the Spruce A timber: namely, transverse expansion, followed by longitudinal expansion. Transverse expansion is chamber extension, while longitudinal expansion is tunnel extension. Transverse expansion is the extension of the nest-gallery by the colony to expand the chamber or explore the surrounding space in the cross-sectional direction perpendicular to the longitudinal axis of the timber, which resulted in a notable increase in volume but not in length. Longitudinal expansion is the extension of tunnel-galleries parallel with the longitudinal axis of the timber, which increased the gallery length.

Half-year data indicated that the volume increased, by 2041 mm^3^ compared to the initial volume (Figure 3b); meanwhile, the length increased 116 mm (Figure 3a). These half-year data correspond with the CT image (Figure 2b), indicating that the group was expanding the nest-gallery to the axial system of the timber, rather than exploring a new area parallel to the longitudinal axis of the timber. The converse pattern was observed for the one-year data, where the length increased by 319 mm from the previous point (Figure 3a) while the volume increased by 1511 mm^3^ (Figure 3b). When the group extended the gallery in the axial direction, the volume increased by 3891 mm^3^ from the previous point (Figure 3b) while the length was extended 149 mm (Figure 3a). In short, the higher the rate of axial expansion, the lower the rate of longitudinal expansion, and vice versa.

### 3.2. Nest-Gallery System in Spruce B Timber

**Figure 4:**
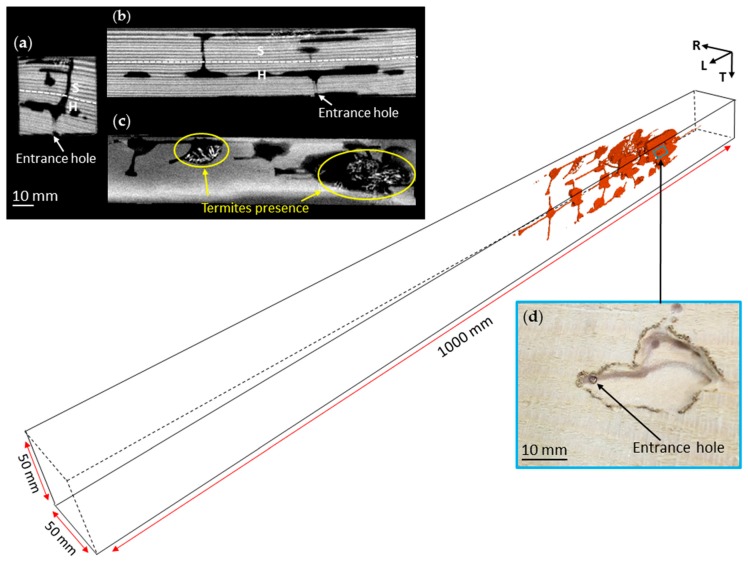
CT images of nest-gallery system in Spruce B timber. (**a**) A 2D cross-sectional image; (**b**) a 2D radial image, indicated that the nest-gallery has been extensively excavated throughout the sapwood (S) and heartwood (H); (**c**) a 2D tangential image, taken from the sapwood part on the opposite surface of the entrance hole; and (**d**) a close-up view of the entrance hole. Termite presence is indicated by an uncolored area inside the nest-gallery in the 3D image or a yellow circular shape in the 2D tangential image. In 2D CT images, the gray value of a pixel corresponds to an index of density: the lighter the color, the denser the area. Springwood and summerwood are indicated by the darker and lighter colors in the images, respectively. The arrow axis represents the longitudinal (L), radial (R), and tangential (T) section of the timber. S, sapwood, H, heartwood.

The nest-gallery system in Spruce B timber is presented in Figure 4. A wide and large cavity (Figure 4d) was excavated by a foraging group of termites on the bottom surface of the timber on the tangential surface of heartwood (Figure 4c). The cavity was surrounded by cement pellets (Figure 4) and was connected to the emergence hole of the natal nest on the attic floor (Figure 2b) when the timber was collected in November 2014. After being separated from its natal colony, the separated group sealed the entrance hole using cement pellets (Figure 4a,b,d). The chambers close to the entrance hole had been widely enlarged from edge to edge of the tangential plane (Figure 4a,c), to accommodate an aggregation of large numbers of termites (Figure 4c). The 2D cross-section and radial CT images suggested that the nest-gallery had been extensively excavated throughout the heartwood and sapwood (Figure 4a,b). The nest-gallery consisted of 32 chambers interconnected by tunnel galleries, and measured 56,613 mm^3^ in volume and 982 mm in length.

### 3.3. Nest-Gallery System in Sugi Timber

**Figure 5:**
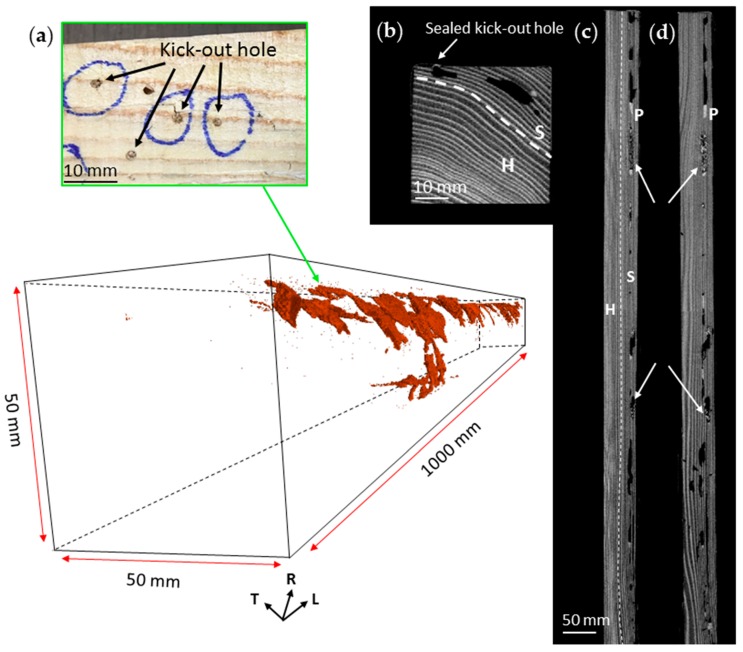
CT images of a nest-gallery system in Sugi timber. (**a**) A close up view of “kick-out holes”, i.e., holes to dispose pellets from the nest-gallery; (**b**) a 2D cross-sectional image; (**c**) a 2D radial image, indicating that the nest-gallery was extensively excavated all over the timber along the sapwood part, parallel to the longitudinal axis of the timber; and (**d**) a 2D tangential image of the sapwood. In 2D CT images, the gray value of a pixel corresponds to an index of density: the lighter the color, the denser the area. Springwood and summerwood are indicated by darker and lighter colors in the images, respectively. The arrow axis represents the longitudinal (L), radial (R), and tangential (T) directions of the timber. S, sapwood; H, heartwood; P, fecal pellets.

Figure 5 displays the nest-gallery system inside a Sugi timber, which shows that the nest-gallery had been excavated all along the timber. The timber was collected in November 2014 and had been located side by side with the Spruce B timber. It is highly possible that the foraging group that attacked the Sugi timber is from the same colony that infested the Spruce B timber, since only one emergence hole was observed from the attic floor (Figure 1b). However, the exact position of the first entrance hole in the Sugi timber could not be located. There were neither spacious entrance holes as found in Spruce A timber (Figure 1a) nor a large cavity as found in Spruce B timber, but rather, numerous holes sealed by cement pellets (Figure 5a). Most of those holes were connected to chambers (Figure 5b), and might have functioned as kick-out holes to dispose pellets out of the nest-gallery. The 2D radial image indicated that the nest-gallery was established parallel to the axial system along the sapwood (Figure 5c), and never extended to the heartwood. The nest gallery measured 114,030 mm^3^ and 1368 mm in volume and length, respectively, with 28 interconnected chambers.

### 3.4. Colony Composition

**Figure 6:**
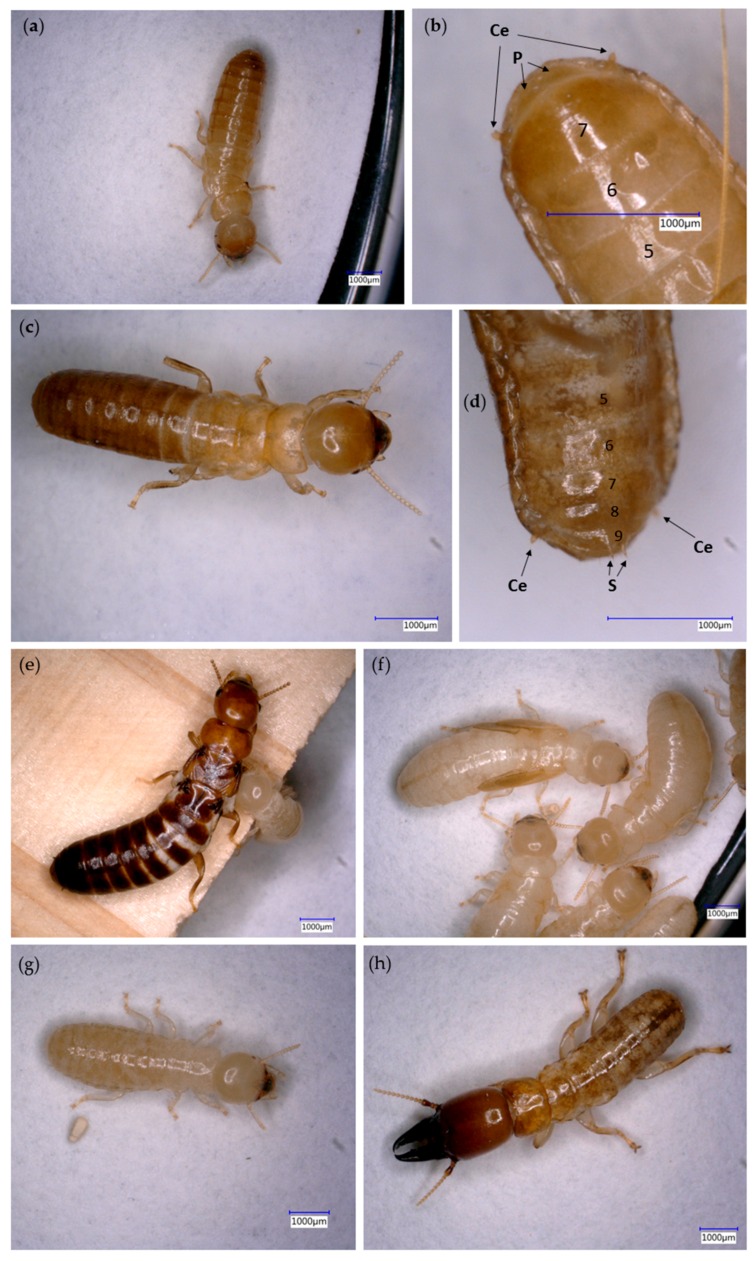
Caste profile of *I. minor* colony. (**a**) A neotenic reproductive emerged from the colony in Spruce A timber and was identified as a (**b**) female neotenic; (**c**) a neotenic reproductive emerged from the colony in Spruce B timber and was identified as (**d**) male neotenic; (**e**) a queen (primary reproductive) from the colony in the Sugi timber; (**f**) nymphs; (**g**) a “false” worker; and (**h**) a soldier. Ce, cerci; P, paraprocts; S, styli. The numbers in Figure 6b,d indicate sternite segments.

The timbers were dissected into 10 pieces (~100 mm/piece) to extract termites and to examine the caste composition. The caste composition of termite colony from each timber is presented in Table 3, while the caste profile is displayed in Figure 6. The total number of termite colonies of Spruce A, Spruce B, and Sugi timbers was 50, 117 and 150, respectively (Table 3). The colony was assessed into castes differentiation as follow:

- *Primary reproductive* (Figure 6e), defined as a reproductive that found a new colony after a nuptial flight [12]. It is characterized by stark sclerotization, the presence of compound eyes and wing marks [13].
- “*False*” *worker* (Figure 6g). The term “false” worker follows Korb et al. [13], and defines a majority of individuals in one-piece nesters [6]. They are not doing tasks and work [14] to the same extent as that of the (true) worker in intermediate and separate nesters [6], i.e., they are less involved in truly altruistic working tasks, such as foraging, brood care, or nest duties. Luykx [15] defined this caste as “*pseudergates*”, since the worker is not a true sterile worker caste; but a totipotent individual [12] that can molt into a secondary reproductive, soldier, or, most commonly, into nymph and alate. They may rather be regarded as large immatures that delay reproductive maturity (‘hopeful reproductives’) [13,16].
- *Soldier* (Figure 6h), a permanently sterile caste, defined as an individual with a strongly sclerotized head with defensive attributes (enlarged mandibles and phragmotic shape) [12].
- *Nymph* (Figure 6f), defined as individual with visible wing buds, which indicates progressive molting into an imago [12,13].
- *Neotenic reproductive* (Figure 6a,c), defined as a secondary reproductive with juvenile morphological characteristics [12], characterized by the absence of wings, the lack of compound eyes, and a cuticle color that is less sclerotized than that of primary reproductives [13].

The majority of individuals within the colony in Spruce A, Spruce B, and Sugi timbers were “false” workers, at 76.0%, 76.1%, and 66.7%, respectively; the next most prevalent were nymphs, at 20%, 17.9%, and 31.4%, respectively (Table 3). Only the colony in Sugi timbers was observed to have a primary reproductive, a queen (Figure 6e), but the presence of a male reproductive could not be located. The colony in both spruce timbers was observed without primary reproductives; however, a neotenic emerged from both colonies to replace the absent reproductives. The neotenic reproductive in Spruce A timber was identified as a female neotenic (Figure 6b), while in Spruce B timber it was a male neotenic (Figure 6d). In female neotenic reproductives, the last abdominal sternite (the seventh, which is enlarged and covers the eighth and ninth sternites) is about as long as it is wide [15], with paraprocts [17,18] observed at the posterior margin (Figure 6b); in males, the last abdominal sternite (the ninth) is only about 1/3 as long as it is wide [14], and styli [17,18] are observed at a posterior margin (Figure 6d). Soldiers were observed in each colony, comprising 2%, 5.1%, and 1.3% of the entire colony in Spruce A, Spruce B, and Sugi timbers, respectively.

## 4. Discussion

By evaluating foraging activities in nest-gallery development, two kinds of foraging behavior are observed: individual foraging and collective foraging. Heidecker and Leuthold [19] suggested that foraging activity is properly called “individual” when it takes place during solitary foraging, while it is “collective” when it is feasible in relation to the group as a colony. Individual foraging was observed in the excavation of tunnel-galleries. This solitary foraging showed wood selectivity; i.e., it was particularly marked in springwood but not summerwood, and was parallel to either the radial or longitudinal axis of the growth ring. This selective foraging may be driven by foraging efficiency [20], by which individuals build efficient tunnels to gain the highest nutritional value [21], and at the same time, maintain its optimum fitness and energy spent. While the concept of foraging efficiency has only been explored in subterranean termites [20], the tunneling activity in drywood termites also shows some attributes of foraging efficiency, indicated by selectivity for the softer part of the growth ring, thereby reducing energy spent on foraging and excavation. This result supports previous evidence [11] showing that the interactions between *I. minor* colonies and wood generate a distinctive and unique foraging pattern, which leads to selective excavation of the nest-gallery system.

Collective foraging was expressed in the excavation of chambers and the whole system of the nest-gallery. Chambers were excavated and enlarged by aggregate individuals working together as a group; thus, labor resources were sufficient to excavate across several annual growth rings. The nest-gallery excavation in spruce timbers was different from that in sugi. In spruce timbers, the nest-galleries were extended all over the sapwood and heartwood (Figure 2 and Figure 5) while, in sugi, the nest-gallery never extended into the heartwood (Figure 5). The heartwood of sugi has been reported to have resistance to termites [22], and its extractives have termicidal [23,24] and anti-termite activities [25]. Therefore, termites in sugi may avoid the heartwood. Even though the heartwood of spruce contains about 70% more extractives than the sapwood [26], the results indicated that the chemicals have low efficacy against *I. minor*. These different results suggested the adaptability of *I. minor* colonies to the timber environment.

Bonabeau [27] characterized social insect colonies as a complex adaptive system (CAS) [28] by six characteristics: (1) dispersed interactions among colony members; (2) decentralized control; (3) hierarchical organization; (4) perpetual reproduction; (5) continual adaptation to changing environmental conditions; and (6) self-organization. These six CAS characteristics can be identified as stigmergic behaviors in social insects. Stigmergy defines a colony as an integrated unit that possesses the ability to process a large amount of information in a distributed manner, such as decisions on task allocations by individuals, coordination of the colony members’ activity, undertaking enormous nest construction works, and also flexibility in response to external and internal challenges [27]. Stigmergic coordinations result from three essential elements: individual autonomy, localized interactions, and autonomous processes selected from individual-colony interaction.

In the present study, stigmergic coordinations were observed in two particular behaviors: sealing a tunnel gallery that ends at the outer edge of the timber (Figure 2a,b); and transporting fecal pellets to particular chambers located beneath timber surface (Figure 2, 3D images). When a termite individual encounters a tunnel exposing the nest to the outer environment (Figure 2a, 2D radial image), this individual information is collected and possibly transferred into the “colony decision” to seal that particular tunnel (Figure 2b) as a part of nest defense and protection. The fact that the sealed tunnel was re-accessable (Figure 2c,d) indicates a dynamic change in the nest-gallery system: self-organization and continual adaptation to a changing environment. Similar fortification behaviors were also encountered by Indrayani et al. [29], as *I. minor* individuals were reported to build a barrier using cement pellets to block their contact with bait-chemicals. Locating the fecal pellets in certain chambers inside the nest-gallery could be explained from the same stigmergic decisional process to maintain the connectivity and network distribution of the nest-gallery. This argument is supported by a previous finding that *I. minor* individuals collected the wood fragments they foraged and carried them to other locations [30], which may be a behavior necessary to maintain the optimum condition of the network of nest-galleries.

The presence of primary reproductives may have an effect on the way the colony forages and establishes the nest-gallery. In spruce timbers where the primary reproductives are absent, termites were observed to aggregate in certain chambers (Figure 2 and Figure 4) and always move together as a group. During extraction, termites were collected from only two or three pieces out of 10 pieces of cut timber. In CT data, they were found to forage and aggregate relatively close to one other; therefore, excavation of the nest-gallery tended to concentrate on certain areas of the timber (Figure 2 and Figure 4). On the other hand, the isolated group with the primary reproductive inside the Sugi timber was found to be more distributed over the entire timber (Figure 5). During extraction of the colony, termites were collected from eight pieces out of the 10 pieces of cut timber.

The presence of a single queen in the *I. minor* colony inside the Sugi timber (Figure 6e) is not an uncommon situation in nature [31]. Since drywood termites’ movement is dynamic, it is highly possible that the colony members may forage separately from the pairing reproductives, and be located in different chambers or even different pieces of timbers. In our case, foraging groups of termites emerged from the attic-floor and attacked the adjacent timber samples. When the timbers were collected, the foraging groups were isolated from their natal nest, which was previously contiguous. This interference may force the groups to adjust their caste compositions by replacing the absent reproductives, as we observed in the isolated groups inside both spruce timbers (Figure 6a,c).

In Kalotermitidae, the tendency of immature stages to become neotenics is called reproductivity [32]. Both neotenics were observed without wing buds, which indicated that they had molted from the pseudergate stage. Our observations support a previous report suggesting that pseudergate reproductivity is generally quite high, at least as high as, or even higher, than those of nymphs and larvae [16]. Interestingly, the sexes of those neotenics were different: female in Spruce A timber (Figure 6b) and male in Spruce B timber (Figure 6d). Previously, we thought that the secondary reproductive first to emerge would be a female reproductive; however, these results suggested that is not always the case. Further study is necessary to better understand this issue.

Spruce A timber was opened three years and four months after collection (Table 2). Based on the CT data (Figure 2a), the timber was newly infested just before collection. Spruce B and Sugi timbers were opened one year and four months after collection (Table 2). However, the CT data suggested that those timbers might have been infested 3–6 months before collection (Figure 4 and Figure 5). An earlier report about the emergence of neotenic reproductives in an orphaned *I. minor* colony was reported by Harvey [10], who assessed isolated groups consisting of 20–30 individuals in laboratory conditions (under 80 °F and RHs 70%). The first evidence of morphological change of the individuals of the sixth instars (equal with first nymphal instar in Roisin’s [12] caste developmental scheme) into supplementary reproductives in his study appeared within four days to one week after orphaning. In three weeks, the eyes became conspicuously pigmented, almost black, and showed distinctive color changing from the mesothorax to the abdomen. At the end of a month these color changes reached their maximum, and then ceased. The orphaned colonies showed that the first egg was laid by the end of the second months, and by the end of the first year, 5–12 progenies ranging from first to fifth instar had emerged.

By examing the color of neotenic bodies in the isolated groups from both spruce timbers and comparing it with Harvey’s description, it seems that the neotenics in those groups had just emerged. The fact that we didn’t find any eggs indicated that the secondary reproductives had not yet reached complete development. Harvey described that secondary reproductives emerged in perfect molt just one month after isolation; however, our case in both spruce timbers indicates a much longer time for the colony to adjust its caste composition. Atkinson [33] reported that seasonal trends in colony composition were relatively weak in *I. minor*. He observed that all or most of the alates in *I. minor* colonies appeared to emerge whenever suitable environmental conditions were present. This might mean that the emergence of replacement reproductives in the isolated groups of *I. minor* in spruce timbers occurred when the groups met “suitable environmental conditions” [33].

Then the major question arises: to what extent does “the suitable environmental condition” facilitate the emergence of reproductives in *I. minor*?

Interesting comparative data regarding reproductive decision-making in another Kalotermitid, *C. secundus*, have been reported by Korb and Lenz [34] and Hoffman et al. [35]. They argued that “nest value” mediated the decision-making of replacement reproductives in wood-dwelling termites. Individuals in *C. secundus* continually evaluate societal conditions (e.g., the absence of an existing royal pair), change their development and induce competition in an attempt to take over a higher nest value. Nest value greatly determines an individual’s potential fitness, which means that a high-value nest provides more potential fitness benefits than a low-value nest. In drywood termites, nest value may relate to wood resources (food and nest space) relative to colony size. As drywood termites have the ability to assess the wood size by vibration [26], the remaining value of the current nest, such as nutritional richness, may be a critical factor in an individual’s decision about whether to develop into a replacement reproductive and take over the nest.

However, Lenz questioned whether reproductive decision-making in *I. minor* had a similar response as that of in *C. secundus* (Lenz and Yoshimura, personal communication). An experiment set up in orphaned *I. minor* colonies using the same ratio model of nest value and colony size, as in *C. secundus*, drove a much slower response in the neotenic production rate. *Incisitermes minor* did not produce the same response as *C. secundus*, suggesting that the model might not fit [36]. This inference contradicts Harvey’s description [10] about the emergence of replacement reproductives in *I. minor* colonies. Further work is necessary to unravel the factors that trigger and regulate neotenic numbers in orphaned *I. minor* groups.

## 5. Conclusions

The documentation of colonization process of foraging groups of *I. minor* in previously unoccupied timbers using X-ray CT has provided better understanding on how isolated groups of *I. minor* develop and maintain the nest-gallery system; as well as to sustain the colony. In establishing nest-gallery, *I. minor* showed selective foraging activities and adaptability to different timber enviroments. Stigmergic behaviors were observed in the way of isolated groups of *I. minor* maintain the nest-gallery system, which was expressed in sealing a tunnel gallery that ends at the outer edge of the timber; and transporting fecal pellets to particular chambers located beneath timber surface. The isolated groups of *I. minor* showed dynamic change in caste composition to sustain the colony. In both groups in which the primary reproductive are absent, a replacement reproductive has emerged from pseudergate stage. However, the sexes of replacement reproductive, time interval and the suitable conditions to facilitate the emergence of replacement reproductive are not been fully understood yet.

## Figures and Tables

**Table 1:** The record of new nest-founding activity by *I. minor*.

Timber Specimens	New Nest-Founding Activity			
	Nuptial Flight (Dealate Reproductives)		Colony Activity	Total
	Successful Nest-Founding *	Attempt **		
Spruce (*n* = 60)	29	21	2	52
Sugi (*n* = 40)	10	8	1	19

* Successful nest-founding was a sealed entrance hole, indicated that the pair of dealates successfully established a royal chamber; ** Attempt was an unsealed entrance-hole or an abandoned entrance-hole without the pair of dealates.

**Table 2:** Nest-founding activity from colony infestation.

Timber			Detail Location of Infestation on Timber				
Specimens	Collected	Opened	Sapwood	Heartwood	Tangential Section	Radial Section	Cross Section
Spruce A	November 2012	March 2016	〇		〇		
Spruce B	November 2014	March 2016		〇	〇		
Sugi	November 2014	March 2016	〇			〇	

**Table 3:** Caste composition of drywood termite colony from each timber.

Timber	Caste Composition					Total
	Primary Reproductive	“False” Worker	Soldier	Nymph	Neotenic Reproductive	
Spruce A	-	38	1	10	1 *	50
Spruce B	-	89	6	21	1 **	117
Sugi	1 ***	102	2	48	-	153

* Female neotenic reproductive (Figure 6a,b); ** male neotenic reproductive (Figure 6c,d); *** queen (Figure 6e).
